# Cross-Species Comparison of the Pan-RAF Inhibitor LY3009120’s Anti-Tumor Effects in Equine, Canine, and Human Malignant Melanoma Cell Lines

**DOI:** 10.3390/genes15020202

**Published:** 2024-02-03

**Authors:** Yu Gao, Eva-Maria Packeiser, Sophia Wendt, Anett Sekora, Jessika-Maximiliane V. Cavalleri, Barbara Pratscher, Moosheer Alammar, Maja Hühns, Bertram Brenig, Christian Junghanss, Ingo Nolte, Hugo Murua Escobar

**Affiliations:** 1 Department of Small Animal Medicine and Surgery, University of Veterinary Medicine Hannover, Foundation, 30559 Hannover, Germany; 2 Department of Medicine, Clinic III, Hematology, Oncology and Palliative Medicine, University Medical Center Rostock, 18057 Rostock, Germany; 3 Clinical Unit of Equine Internal Medicine, Department for Companion Animals and Horses, University of Veterinary Medicine Vienna, 1210 Vienna, Austria; 4 Clinical Unit of Small Animal Internal Medicine, Department for Companion Animals and Horses, University of Veterinary Medicine Vienna, 1210 Vienna, Austria; 5 Institute of Pathology, University Medicine of Rostock, Strempelstrasse, 18055 Rostock, Germany; 6 Institute of Veterinary Medicine, Division of Molecular Biology of Livestock and Molecular Diagnostics, Georg-August-University of Göttingen, 37077 Göttingen, Germany

**Keywords:** equine malignant melanoma, canine malignant melanoma, Pan-RAF inhibitor, LY3009120, DNA sequencing, genetic evaluation, cellular response

## Abstract

Malignant melanomas (MMs) are the abnormal proliferation of melanocytes and are one of the lethal skin cancers in humans, equines, and canines. Accordingly, MMs in companion animals can serve as naturally occurring animal models, completing conventional cancer models. The common constitutive activation of the MAPK and PI3K pathways in MMs has been described in all three species. Targeting the related pathways is considered a potential option in comparative oncologic approaches. Herein, we present a cross-species comparative analysis exposing a set of ten melanoma cell lines (one human, three equine, and six canine) derived from primary tumors or metastasis to a pan-RAF and RAF dimer inhibitor (LY3009120). Cellular response (proliferation, biomass, metabolism, early and late apoptosis/necrosis, and morphology) and the presence of pathogenic single-nucleotide variants (SNVs) within the mutational hotspot genes *BRAF* exon 11 and 15, *NRAS* exon 2 and 3, *KRAS* exon 2, and *KIT* exon 11 were analyzed. This study showed that equine malignant melanoma (EMM) cells (MelDuWi) harbor the *KRAS* p.Q61H mutation, while canine malignant melanoma (CMM) cells (cRGO1 and cRGO1.2) carry *NRAS* p.G13R. Except for EMM metastasis cells eRGO6 (wild type of the above-mentioned hotspot genes), all melanoma cell lines exhibited a decrease in dose dependence after 48 and 72 h of exposure to LY3009120, independent of the mutation hotspot landscape. Furthermore, LY3009120 caused significant early apoptosis and late apoptosis/necrosis in all melanoma cell lines except for eRGO6. The anti-tumor effects of LY3009120 were observed in nine melanoma cell lines, indicating the potential feasibility of experimental trials with LY3009120. The present study reveals that the irradiation-resistant canine metastasis cells (cRGO1.2) harboring the *NRAS* p.G13R mutation are significantly LY3009120-sensitive, while the equine metastases-derived eRGO6 cells show significant resistance to LY3009120, which make them both valuable tools for studying resistance mechanisms in comparative oncology.

## 1. Introduction

In humans, melanoma is usually associated with pigmentation, fair skin, exposure to ultraviolet radiation, and lethality. Aberrations in kinases such as proto-oncogene *BRAF* (exons 11 and 15; p.V600E), *NRAS* (exons 2 and 3; codons 12, 13, and 61), or *KIT* (exons 11, 13, 17, and 18) are key factors in human skin melanomas, regulating melanocyte proliferation survival and apoptosis. *BRAF* and *NRAS* mutations have been reported in 41% and 18% of human cutaneous melanomas, respectively, and *KIT* mutations in 8.6–25% of acral/mucosal/chronically sun-damaged skin melanomas [1,2]. In contrast, canine melanomas are mostly oral MMs also harboring *KIT* mutations. Equine melanomas mostly occur as melanocytomas or dermal melanomas in grey horses. Thereby, *STX17* and *DPF3* mutations represent inherited aberrations of oncogenic candidate genes [3]. Equine and canine melanoma exhibit fewer UV-induced melanoma characteristics and lower mutational hotspot frequencies compared to humans. However, despite the lower frequencies, *BRAF*, *NRAS*, *KRAS*, and *KIT* are somatic SNVs identified in canine melanomas, accounting for 1.8% (5/272), 8.0% (25/311), 13.1% (27/206), and 22.9% (16/70), respectively. In horses, *BRAF*, *NRAS*, *KRAS*, and *KIT* mutations have been observed in 7.1% (2/28), 8.3% (5/60), 0/62 (0%), and 3.6% (1/28) of melanoma samples (summary see Supplement Table S1) [4,5,6,7]. Studies have shown that *NRAS*, *PTPRJ*, and *TP53* are the most significant known mutated genes in canines. These genes have been reported as pathogenic melanoma drivers in both humans and dogs [7,8]. The exact elucidation and comparison of the mechanisms acting in MMs of the three species are key to evaluating if targeted interventions have the potential to be transferred between species. Multiple carcinogenic pathways, particularly, the overactivation of the MAPK and PI3K signaling pathways, are closely associated with the pathogenesis of human, canine, and equine melanomas with high levels of activated ERK1/2 [4,8,9]. The MAPK pathway is affected in 43% of canine oral melanoma cases, suggesting that potentially significant driver mutations may also be present [8]. The shared genetic profile between human and canine malignant melanomas underscores the potential utility of canines as preclinical models for testing melanoma treatments destined for use in humans [6,7,10]. Furthermore, part of the clinical characteristics of melanomas are shared within all three species. Also, partial characteristics of melanocytes in pathology, tumorigenesis pathway enrollment, and genetic background are similar (Supplement Table S2) [10,11,12].

Surgical resection is the most preferred choice as a local tumor treatment for human, canine, and equine melanomas at an early stage [13]. The treatment options for advanced (metastatic or unresectable) melanoma are complex, with chemotherapy and antiangiogenic therapy as the traditional systemic therapies. Therefore, in recent years, targeted therapy and immunotherapy have emerged as the preferred options for the systemic therapy of human advanced melanomas and metastasis [14,15]. This is primarily due to their ability to target specific molecules or pathways involved in the growth and progression of melanomas in humans as well as the high mutational burden often detected in tumors. Clinically approved tyrosine kinase inhibitors (TKIs) such as toceranib, masitinib, and imatinib have shown significant effects in the treatment of canine mast cell tumors carrying the *KIT* mutation, outperforming the former gold-standard chemotherapy treatment based on vinblastine sulfate (VBL) [16]. However, no targeted drugs are currently approved for horse cancers. Two compounds may be potential candidates, the Kunitz-type protease inhibitor (Amblyomin-X) and the p38 MAPK inhibitor (Doramapimod) [17,18], showing promising results in equine tumors.

LY3009120 (DP-4978) is a potent pan-RAF and dimer inhibitor that targets multiple serine/threonine-specific protein kinase family members, including A-Raf, B-Raf, and C-Raf. The kinases represent key components of the oncogenic-acting Ras-Raf-MEK-ERK signal transduction cascade pathway. In general, activated receptor tyrosine kinases (RTKs) in the plasma membrane transduce signals to activate Ras-GTP by binding GRB2 and recruiting Son-Of-Sevenless (SOS), stimulating and mediating guanine nucleotide exchange factors (GEFs) and the GTP loading of Ras GTPases (KRAS, NRAS, and HRAS). Downstream signaling of the RAF kinase family members is activated and catalyzes the phosphorylation and activation of MEK1 and MEK2, in turn, activating extracellular signal-regulated kinase (ERK) [19]. Compared to certain first-generation BRAF monomer-selective inhibitors, LY3009120 shows higher binding potencies to all RAF isoforms. This includes heterodimers and homodimers of BRAF or CRAF [20]. This binding effectively inhibits the activity of the dimer, leading to the suppression of downstream signaling such as the phosphorylation of MEK and ERK. This mechanism results in the cascade inhibition of signals that regulate cell growth and proliferation. In addition, the application of certain first-generation BRAF monomer-selective inhibitors leads to acquired resistance in *BRAF* wild-type (WT) cells due to the increased level of Raf dimerization. For example, Vemurafenib and dabrafenib, highly selective FDA-approved inhibitors targeting mutant *BRAF*^V600E^ (treatment of metastatic and unresectable melanoma harboring V600E mutations) induce in *BRAF*^WT^ cells the paradoxical activation of the MAPK kinase pathway and promote the growth and metastasis of tumor cells with amplified RAS signals. Currently, three major hypotheses explain this observed resistance. First, the increased level of Raf dimerization due to the presence of a BRAF monomer-selective inhibitor has been suggested to increase the affinity levels between the protomers. Second, the RAF inhibitor insufficiently binds to both protomers, resulting in negative cooperativity (NC) which reduces the affinity of the inhibitor to bind the unoccupied protomer. Therefore, the unoccupied protomer is still activated. Third, RAF inhibitors have been suggested to modulate the RAF autoinhibitory conformational regulation by shifting the balance between both conformations to an active form that is able to dimerize [19,21]. The proposal of the pan-RAF inhibitor (PRi) is an attempt to address this resistance problem due to its special properties of stabilizing the DFG-out/αC-in states of target kinase conformation [20]. Consequently, LY3009120 effectively suppresses the activity of active RAF dimers and suppresses ERK signaling, minimizing the risk of inducing paradoxical activation. In addition, LY3009120 inhibits not only *BRAF*^V600E^ but also *BRAF*^WT^ and *CRAF*^WT^ cells and has demonstrated impressive anti-tumor efficacy during *in vitro* and *in vivo* experiments. The compound has been reported to inhibit the growth of the human melanoma cell line A375 *in vitro* by inhibiting BRAF V600E kinase activity [20]. In addition, LY3009120 has been broadly applied in tumor cells or colorectal carcinoma xenograft models harboring oncogenic *RAS* or *BRAF* mutations and Vemurafenib-resistant melanoma cells as well as tumor cells with oncogenic *BRAF* deletions [19,20,21,22,23,24,25]. LY3009120 or combinations with CDK4/6-inhibitors such as abemaciclib have shown stronger anti-tumor effects with *BRAF*-mutant cancer cells than cancer cell lines with various *NRAS* genotypes [22]. Furthermore, LY3009120 has been reported to inhibit phospho-MEK and ERK signaling in *BRAF*^WT^ cell lines showing an antiproliferation effect in HCT-116 colon cancer cells and significant xenografts (*KRAS*^mut^) regression [25]. 

To summarize, when comparing human melanoma with equine and canine primary or metastatic MMs, it is evident that the relationship between genetic background and targeted therapy in humans is better understood. The testing of LY3009120 can probably target distinct biological melanoma forms across all three species. Further, cross-comparative analyses bear the potential to advance human targeted therapy.

## 2. Materials and Methods

### 2.1. Primary Culture and Melanoma Cells Samples 

Human melanoma cell line A375 (*BRAF* V600E) was purchased from the American Type Culture Collection (ATCC). Equine and canine melanoma cells (details in Table 1) were provided by the cell culture stock of the Clinic for Horses and the Small Animal Clinic, University of Veterinary Medicine Hannover, Foundation, Germany (MelDuWi, TiHoDEpiMel1268 (Mel1268), and TiHoDEpiMel0910 (Mel0910)), and the University of Veterinary Medicine in Vienna, Austria (cRGO1, cRGO1.2, cRGO4, cRGO6, eRGO1, and eRGO6). More details on canine cell lines (cRGO1, cRGO1.2, and cRGO4) characterization can be found in published reference [26]. The morphology aspects of all human, canine, and equine melanoma cell lines are shown in Supplement Figure S1. The A375’s culture method followed the ATCC product sheet. The canine and equine melanoma cell lines’ culture methods followed articles [26,27]. All cells were grown in DMEM high glucose supplemented with 10% characterized fetal bovine serum and 1% penicillin-streptomycin at 37 °C, 5% CO_2_, and 95% humidity.

### 2.2. Inhibitor

LY3009120 (DP-4978) was purchased from Hycultec GmbH (Beutelsbach, Germany). Dimethyl sulfoxide (DMSO) was selected as the solvent, and the final concentration of LY3009120 was configured into a stock solution with 10 mM. The stock solution was stored at −80 °C and configured to the corresponding working concentration before each experiment.

### 2.3. Sequencing

Initially, PCR (according to Table 2) and Sanger sequencing on nine cell lines (MelDuWi, eRGO1, eRGO6, Mel1268, Mel0910, cRGO1, cRGO1.2, cRGO4, and cRGO6) were performed for analyses. In cases of unsuccessful exon amplification or Sanger sequencing, whole exome sequencing (WES) was performed.

#### 2.3.1. DNA, RNA Extraction, and cDNA Synthesis

Cell pellets of A375, MelDuWi, eRGO1, eRGO6, Mel1268, Mel0910, cRGO1, cRGO1.2, cRGO4, and cRGO6 were subjected to gDNA extraction (NucleoSpin^®^ Tissue Kit, Macherey-Nagel GmbH, Dueren, Germany) according to the manufacturer’s instructions.

Cell pellets of Mel1268, Mel0910, cRGO1, cRGO1.2, cRGO4, and cRGO6 canine melanoma cells were resuspended in QIAzol Lysis Reagent (Qiagen, Venlo, The Netherlands) for RNA isolation with an miRNeasy Mini Kit (Qiagen GmbH, Hilden, Germany) according to the manufacturer’s instructions and stored at −80 °C.

The PrimeScript™ RT Reagent Kit (Takara Bio Europe, Saint-Germain-en-Laye, France) was used for cDNA synthesis according to the manufacturer’s instructions.

#### 2.3.2. PCR and Sanger Sequencing

For Sanger sequencing PCR, the amplification of the canine and equine *BRAF* exons 11 and 15, *NRAS* exons 2 and 3, and *KRAS* exon 2 was performed. Information for the three gene fragments’ primers is shown in Table 2. All the primers were purchased from Eurofins Genomics (GmbH; Ebersberg, Germany). The optimal annealing temperatures were determined by means of a gradient PCR program (53 to 63 °C for 35 cycles) using a C1000 Touch™ Thermal Cycler (Bio-Rad Laboratories, Inc., California City, CA, USA). DNA fragments were amplified using a touch-down PCR program (annealing at 53 °C to 63 °C for 7 cycles followed by annealing at 55 °C or 60 °C for 37 cycles) in a 25 µL reaction mixture (Bioron, GmbH, Römerberg, Germany) using 17.65 µL dH_2_O, 2.5 µL 10 × buffer (1× buffer = 10 mmol/L), 50 ng DNA, 100 mmol/L, 2.5 µL dNTPs, 25 mM, 0.75 µL MgCl_2_, 50 pmol/µL primer, and 2.5 U Polymerase 0.1 µL.

The PCR products were recovered by means of 2.5% agarose gel electrophoresis and gel clean-up (NucleoSpin^®^ Gel and PCR Clean-up kit, Macherey-Nagel GmbH, Dueren, Germany).

Subsequently, sequencing reactions were performed with a BigDye Terminator v3.1 Cycle Sequencing Kit (Applied Biosystems, Darmstadt, Germany) with each pair of forward and reverse primers (Table 2), followed by analysis on a 3500 Genetic Analyzer (Applied Biosystems). The sequence data were compared with reference sequences (CanFam3.1 and EquCab3.0 and Table 2) using SeqScape Software v2.7 (Applied Biosystems) and Chromas (v2.6.6).

#### 2.3.3. Whole Exome Sequencing

The DNA samples were sheared to approx. 500 bp using Covaris S220 Focused-ultrasonicator (Covaris Ltd., Brighton, Woodingdean, UK) and then concentrated with Sartorius VIVACON 500 (Fisher Scientific, Thermo, Waltham, MA, USA). Average fragment sizes were analyzed on an Agilent 2100 Bioanalyzer using the DNA 7500 Kit (Agilent Technologies, Santa Clara, CA, USA). Sequencing libraries were generated using a ThruPlex DNA-Seq Kit (Takara Bio Europe SAS, Saint-Germain-en-Laye, France). The libraries were checked again before sequencing on an Agilent 2100 Bioanalyzer using the DNA 7500 Kit. The libraries were processed on a High Output Flow Cell v2.5 (Illumina, San Diego, CA, USA) with 300 cycles using HiSeq2500 (Illumina, San Diego, CA, USA ).

All the FASTQ files assessing the sequencing quality were controlled using the tool FastQC (v0.12.1) before the tool bwa-mem2 (v2.2.1) was chosen for aligning the FASTQ files to the reference genome (CanFam3.1 or EquCab3.0) and, finally, importing the generated BAM or SAM file into IGV (v2.16.1) for a visualization analysis.

### 2.4. Cell Counting and Viability Assays

MM cell lines with a density of 2.0–6.7 × 10^4^ cells/mL were inoculated in 24-well plates and 96-well plates, respectively. LY3009120 was diluted in DMEM medium at different gradient concentrations (range from 0.01 to 10 µM) for A375, MelDuWi, Mel1268, and Mel0910 and a narrow range gradient concentration (range from 0.1 to 5 µM) for the remaining cell lines in cell proliferation, metabolic activity, biomass, and apoptosis/necrosis. After 24 h of incubation, the medium was discarded, and the DMEM medium of varying concentrations of LY3009120 or vehicle DMSO control was added. The exposed cells were incubated at 37 °C with 5% CO_2_ for 48 or 72 h. Assessments of cell proliferation, metabolic activity, cell biomass, and early apoptosis and late apoptosis/necrosis were conducted at designated time points, i.e., 48 and 72 h, in at least three biologically independent replicates.

#### 2.4.1. Proliferation

Proliferation was assessed using trypan blue (Sigma-Aldrich Chemie GmbH, Steinheim, Germany) staining to determine the absolute cell counts. MM cells were harvested and washed with 1× PBS (PAN-Biotech) from 24-well plates after being exposed for 48 or 72 h to LY3009120, the cell pellets were resuspended, stained, and counted using a hemocytometer. Cell proliferation was expressed as the percentage of viable cells treated with the inhibitor when compared to a 100% DMSO control.

#### 2.4.2. Metabolic Activity

Water-soluble tetrazolium-1 (WST-1) can be used to evaluate MM cells’ metabolic activity. After exposure to LY3009120 in a 96-well plate, 15 µL WST-1 (Roche, Mannheim, Germany) was added to the well and incubated for 2 h. Absorbance at 450 nm and 620 nm reference wavelengths was measured using a Multimode Reader (Promega GloMax®-Multi Microplate, Madison, WI, USA). Metabolic activity was expressed as a percentage of the LY3009120-treated group to the DMSO-treated control (control = 100%). During data analysis, a background correction was performed by subtracting the absorbance value of the reference wavelength from that of the corresponding sample’s wavelength. Subsequently, the blank value was subtracted from this corrected difference, ensuring accurate measurements. The resulting value was then correlated with the metabolic activity of the viable cells, providing insight into their functional state.

#### 2.4.3. Biomass Quantification

MM cells’ biomass quantification was evaluated by means of crystal violet (CV) staining. Following the respective 48 h and 72 h incubation periods with the corresponding LY3009120, the medium was discarded. Subsequently, the cells in the 96-well plate were washed once with PBS and fully stained with a 50 µL 0.2% CV solution for a 10 min incubation on a shaker table at room temperature. To carefully elute unbound CV, the cells were washed twice with 200 µL of PBS solution. The plate was dried overnight before quantification, and 100 µL of 1% sodium dodecyl sulfate (SDS) was added to each well while being shaken at room temperature for 10 min. Finally, absorbance measurements were taken at 560 nm with a reference wavelength of 450 nm using the Promega GloMax^®^-Multi Microplate Multimode Reader. In each experimental group, the absorbance of the pure medium was subtracted from the uniform background. The CV value obtained was directly proportional to the cell biomass. Cell biomass was expressed as a percentage when the LY3009120-treated group compared to the DMSO-treated control (control = 100%).

### 2.5. Early Apoptosis and Late Apoptosis/Necrosis Measurements

Early apoptosis and late apoptosis/necrosis were evaluated using flow cytometry FACSVerse™ (Becton, Dickinson, and Company) after Annexin V FITC (Becton, Dickinson and Company, Heidelberg, Germany) and Propidium iodide (PI) (Sigma-Aldrich Chemie GmbH) double staining. The cells that had been exposed to LY3009120 were harvested and washed with PBS after centrifugation at 180× *g* for 10 min at 4 °C. The cell pellet was then resuspended in 100 µL of binding buffer (1×) (Becton, Dickinson, and Company), and 5 µL Annexin V FITC was added and incubated at room temperature for 15 min while protected from light. Thereafter, flow cytometry was performed immediately after adding 400 µL of binding buffer and 15 µL of PI (20 μg/mL) to the cells. Unstained cells and single-stained cells were also determined as the control group. Annexin-/PI- cells were considered living cells. Annexin+/PI- cells were considered early apoptotic cells, and Annexin+/PI+ cells were considered late apoptotic/necrotic cells. Data were analyzed using BD CellQuest™ Pro v6.1 (Becton, Dickinson and Company, Heidelberg, Germany)

### 2.6. Examination of Cell Morphology Changes

MM cells were cultured in 24-well plates and exposed to either LY3009120 (0.1 µM or 1 µM) or DMSO. Subsequently, Pappenheim staining was performed after 48 and 72 h of exposure. To prepare the samples, 1 × 10^5^ cells in 400 µL were harvested and fixed on two Cytoslides using the Shandon Cytospin 3 centrifuge. After air-drying, the slides were immersed in May–Grünwald solution for precisely 6 min, followed by washing with buffer (pH = 7.2) and staining with a Giemsa solution (1:10) for 20 min. After another round of buffer washes, the slides were air-dried. The morphology of the cells was examined and visualized under the Evos XL Core Imaging System (Life Technologies, Darmstadt, Germany) at a magnification of 1000 times. Each experiment was replicated three times to minimize random errors.

### 2.7. Statistical Analyses

Each experiment was meticulously executed with a minimum of three independent biological replicates. The results encompassing cell proliferation, metabolic activity, biomass quantification, and apoptosis/necrosis were presented as the mean value accompanied by the standard deviation (SD). To determine statistical significance, we performed one-way ANOVA using GraphPad Prism 8 (meeting a Gaussian distribution within each group). The level of statistical significance was precisely indicated with * for *p* < 0.033, ** for *p* < 0.002, and *** for *p* < 0.001, elucidating the magnitude of significance in comparison to the control group.

## 3. Results

### 3.1. The Genotypes of BRAF, NRAS, KRAS, and KIT Mutation in Ten Melanoma Cell Lines

The wild type and mutant of four gene loci (*BRAF exon 11 and 15*, *NRAS exon 2 and 3*, *KRAS exon 2*, and *KIT exon 11*) were evaluated in ten cell lines, including human (A375), canine (Mel1268, Mel0910, cRGO1, cRGO1.2, cRGO4, and cRGO6), and equine lines (MelDuWi, eRGO1, and eRGO6) (Table 3). All the canine and equine melanoma cell lines detected *BRAF* exon 15 by means of Sanger sequencing or WES. All the canine and equine melanoma cell lines retained their wild-type status of *BRAF* exon 15 and 11, *NRAS* exon 3, and *KIT* exon 11 according to WES (Table 3). From the Sanger sequencing results, the p.Q61H mutation of *KRAS* exon 2 was found in the MelDuWi (CAA>CAT, p.Gln61His), while the primary cRGO1 exhibited the p.G13R mutation in *NRAS* exon 2. The results from whole exome sequencing show that cRGO1 and the metastasis cRGO1.2 both exhibited the p.G13R mutation (Codon 13, GGT>CGT, p.Gly13Arg) in *NRAS* exon 2. Worth mentioning is that cRGO1.2 originated from the same canine oral melanoma as cRGO1, while metastasis cRGO1.2 harbored resistance to irradiation.

### 3.2. Application of Pan-RAF Inhibitor (LY3009120)

#### 3.2.1. Effect on Viability and Proliferation

**Figure 1:**
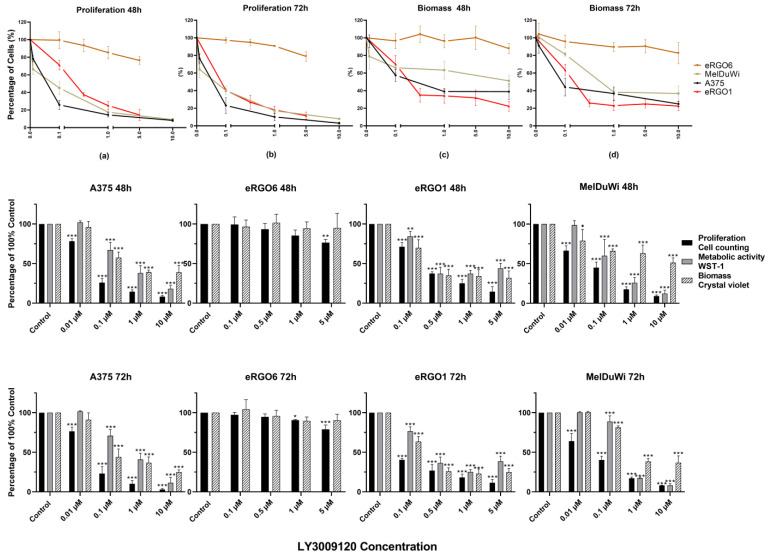
Proliferation, biomass, and part of the metabolic activity changes in A375 and all equine cell lines. Subfigures (**a**–**d**) are line graphs of proliferation and biomass changes in A375 and all equine cell lines after exposure to different concentrations of LY3009120 in 48 and 72 hours. Proliferation is evaluated with trypan blue staining. Biomass is evaluated by means of crystal violet staining, and metabolic activity is evaluated with a WST-1 assay. The significance of a treatment effect compared to the DMSO control is determined by means of a one-way ANOVA and is displayed as *: *p* < 0.033, **: *p* < 0.002, and ***: *p* < 0.001 (*n* ≥ 3).

**Figure 2:**
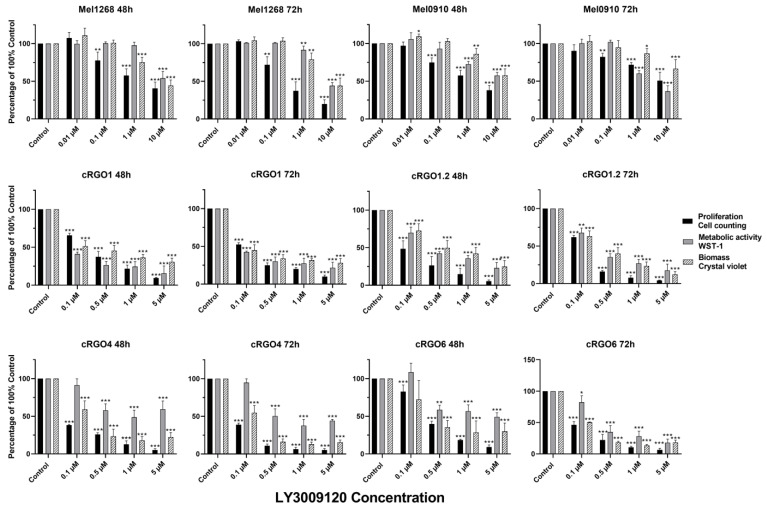
Proliferation, biomass, and metabolic activity changes in all canine cell lines. Proliferation is assessed by means of trypan blue staining. Biomass was evaluated with crystal violet staining, and metabolic activity was evaluated via a WST-1 assay. The significance of a treatment effect compared to the DMSO control is determined by means of a one-way ANOVA and is displayed as *: *p* < 0.033, **: *p* < 0.002, and ***: *p* < 0.001 (*n* ≥ 3).

Overall, LY3009120 acted in a dose-dependent and time-dependent manner in all *RAF/RAS* mutant and wild-type cell lines, invariably decreasing to varying degrees, except for liver metastasis equine melanoma cells eRGO6. The observed effects showed no significant difference between the 48 and 72 h except for cRGO6, which showed a slightly increased effect at the 72 h time point when LY3009120 was above 0.5 μM. LY3009120 ≥0.5 μM was found to be significantly effective against cRGO1.2, cRGO4, cRGO6, cRGO1, eRGO1, and MelDuWi (Figure 1 and Figure 2). Furthermore, cRGO1.2, cRGO4, cRGO6, and eRGO1, in terms of reduced biomass and metabolic activity effect at 1 μM of LY3009120, showed high inhibition, as did the positive control A375 (Figure 1, Figure 2 and Figure S2).

In general, when comparing the mutant to the wild type, *RAF/RAS* mutant cell lines are more sensitive to LY3009120 than the wild type during metabolic activity. In our study, for the wild type, a significant difference from the control was considered effective, with a 48/72 h effective concentration of LY3009120 at ≥1 μM in the canine cell lines (Figure 2). Regarding the *RAF/RAS* mutant in all three species, the 48/72 h significant effective concentration of LY3009120 was ≥0.1 μM for cell viability and antiproliferation (Figure 1 and Figure 2).

In terms of proliferation and biomass, A375 reduced by 89.86% and 63.28%, respectively, at 1 μM for 72 h. Among three equines MM cell lines (Figure 1), eRGO6 (wild type of *BRAF*, *NRAS*, and *KRAS*) showed a low sensitivity or was even resistant with respect to proliferation and biomass. eRGO1 (wild type of *BRAF*, *NRAS*, and *KRAS*) and MelDuWi (*KRAS* p.Q61H mutation) showed significant antiproliferative and biomass-reducing responses. The cell proliferation of MelDuWi and eRGO1 decreased by 82.88% and 81.87%, and their biomass decreased by 75.02% and 61.69%, respectively, in 1 μM (72h). The eRGO6 was the least sensitive and most resistant among all three species’ cell lines, even when tested at a high concentration of 5 μM for 72h, showing the reduction of the cell proliferation and biomass by only 21.01% and 9.60%, respectively. In canine MM cell lines (Figure 2), cRGO1.2 (carrying *NRAS* p.G13R mutations) and wild-type cRGO4 exceeded the positive control A375 with respect to reductions in cell proliferation and biomass at a 1 μM concentration, with the largest reductions in cell proliferation being 95.73% and 94.82% and, in biomass, 87.76% and 84.39%. Relative to cRGO1.2 and cRGO4, the sensitivities of the remaining canine MM cell lines to LY3009120 exposure were in between, with cRGO6, cRGO1, Mel1268, and Mel0910 in descending order. The proliferation of cRGO6, cRGO1, Mel1268, and Mel0910 was reduced by up to 93.52%, 90.58%, 80.09%, and 61.91% at the highest tested concentrations of 5 or 10 μM, respectively. The biomass of cRGO6, cRGO1, Mel1268, and Mel0910 was reduced by up to 81.79%, 69.30%, 55.75%, and 42.15% at the highest tested concentrations of 5 or 10 μM, respectively.

In metabolic activity, there was a significant decrease in A375 of 59.23% at 1 μM and 88.51% at 10 μM for 72 h. The 72 h exposure measurements of MelDuWi showed the strongest inhibition, followed by cRGO1.2, with the largest reductions of 91.78% and 82.38%, respectively (Figure 1). The metabolic activity of the rest of the cell lines (cRGO1, cRGO6, eRGO1, Mel0910, cRGO4, Mel1268, eRGO6 (72 h), and eRGO6 (48 h)) was reduced by up to 84.27%, 82.06%, 74.82%, 63.38%, 55.92%, 55.73%, 50.27%, and 13.2% at the highest tested concentrations of 5 or 10 μM, respectively. It is worth mentioning that, in metabolic activity, eRGO1 and cRGO4 showed a rebound after the high concentration of 1 μM for 72 h, with the largest rebounds being 13.40% and 6.42%, respectively (Figure S2).

#### 3.2.2. Application of Pan-RAF Inhibitor (LY3009120) Induces Early Apoptosis and Late Apoptosis/Necrosis of Melanoma Cell Lines

**Figure 3:**
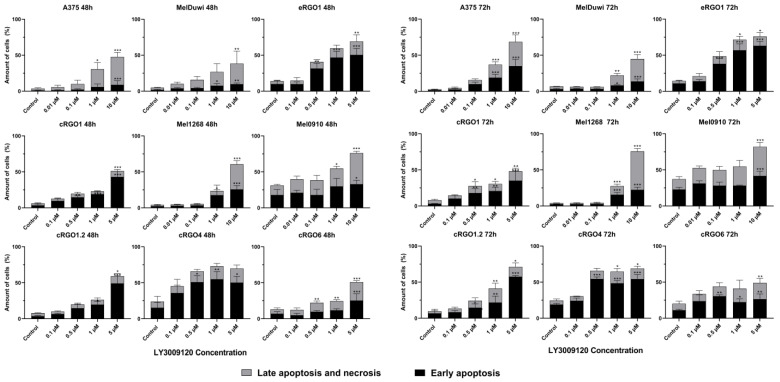
Induction of early and late apoptosis/necrosis in human, equine, and canine melanoma cell lines after 48 and 72 h incubation with LY3009120 identified via Annexin V FITC and flow cytometry by means of propidium iodide (PI) double staining. The significance of a treatment effect compared to the DMSO control is determined via a one-way ANOVA and displayed as *: *p* < 0.033, **: *p* < 0.002, and ***: *p* < 0.001 (*n* ≥ 3).

The results showed that an increased concentration of LY3009120 was associated with the increased apoptosis of cell lines. When the concentration of LY3009120 reached 5 μM or 10 μM, except for eRGO6, all cell lines exposed to the inhibitor for 72 h showed the significant increase in inducted apoptosis/necrosis. When the LY3009120 concentration was 1 μM (72 h), A375, cRGO1, cRGO1.2, cRGO4, cRGO6, eRGO1, MelDuWi, and Mel1268 began to show significant induction of early apoptosis and late apoptosis/necrosis (Figure 3).

#### 3.2.3. Morphological Changes in Melanoma Cell Lines with Application of Pan-RAF Inhibitor (LY3009120)

**Figure 4:**
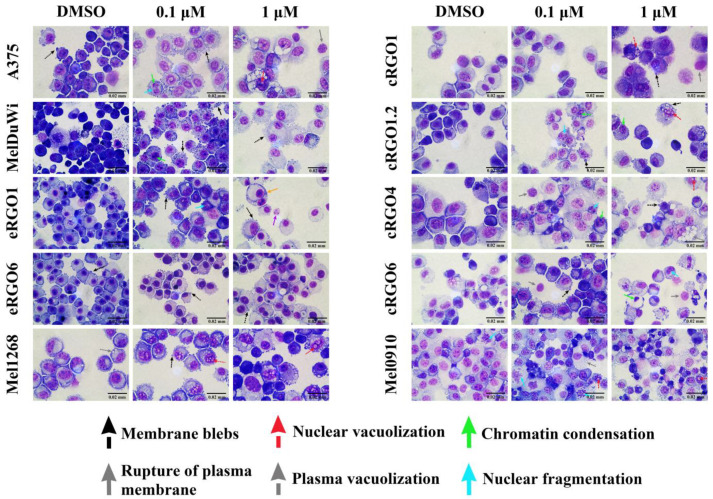
Morphological changes in ten melanoma cell lines with the application of LY3009120 by means of Pappenheim staining. Magnification: 1000×. Ten human, canine, and equine melanoma cell lines after DMSO or LY3009120 inhibitor exposure for 48 or 72 h. Melanin is observed in the cytoplasm of MelDuWi (solid black arrow in the upper right of the 0.1 μM). These indicators include the presence of membrane blebs (black arrows), numerous nuclear vacuoles (red arrows) and plasma vacuoles (grey arrows), chromatin condensation (solid green arrow), nuclear fragmentation (karyorrhexis, solid blue arrow), cellular fragmentation (solid purple arrow), rupture of the plasma membrane (solid grey arrow), phagocytosis (solid orange arrow), and marked distortions in cellular morphology. Except for eRGO6, comparable morphological changes are observed in all the samples treated with LY3009120 at the concentration above 0.1 μM. More pronounced evidence is observed in A375, cRGO1.2, cRGO4, eRGO1, cRGO6, Mel1268, and MelDuWi.

The cell morphology of the ten cell lines was thoroughly investigated by means of Pappenheim staining at a magnification of 1000× and in comparison with DMSO control samples (Figure 4). All the cell lines, except for eRGO6, exposed to the studied medication displayed clear indications of apoptosis and cellular stress, beginning with the progressive condensation of the plasma material, the formation of bubble-like bulges on the cell surface, and apoptotic bodies and ending with the fragmentation of the cells. However, the decrease in the cell number was severe and consistent with increases in the dose in cell lines cRGO1.2, cRGO4, eRGO1, cRGO6, A375, and MelDuWi, with no significant difference between the 48 and 72 h time points. These changes were observed in all cell lines except for eRGO6, which showed no evidence of drug interference compared to the control.

## 4. Discussion

Across three species (human, canine, and equine), the RAS-RAF-MEK-ERK signaling cascade and its associated proto-oncogene mutations are involved in melanoma pathogenesis [4,7,9,29]. In the herein-presented compound evaluation, ten MM cell lines were analyzed in total, with one human (A375), six canine (Mel1268, Mel0910, cRGO1, cRGO1.2, cRGO4, and cRGO6), and two equine cell lines (MelDuWi and eRGO1) showing significant growth inhibition after being exposed to LY3009120 (0.1–5 μM or 0.01–10 μM) for 48 h and 72 h. Further, these cell lines showed specific responses in several cell biological parameters such as proliferation, biomass, metabolism, early and late apoptosis/necrosis, and morphology when exposed to LY3009120. Interestingly, the metastasis-derived eRGO6 represented an exception to the general observed effects. Further, equine and canine MM cell lines bearing the *RAS* mutant responded in a more sensitive manner to LY3009120 compared to the wild-type cell lines, when analyzing metabolic activity. Interestingly, one of the metastatic EMM cell lines (eRGO6, wild type for *BRAF*, *NRAS*, *KRAS*, and *KIT*) revealed resistance to LY3009120, while the metastatic canine OMM cell line (cRGO1.2, *NRAS* p.G13R) showed a significant sensitivity to compound exposure.

In comparison to selective BRAF^V600E^ inhibitors, LY3009120 has shown inhibitory effects in a wider range of tumor cells including *RAF*/*RAS*^mut^ and WT *in vivo* and *in vitro* [17,18,19,20,21,22,23,24,25]. Indeed, selective BRAF^V600E^ inhibitors have been proven to induce RAF dimerization and activate ERK signaling through increased RAS activity, thereby increasing growth in *RAF*^WT^ tumors [30]. Furthermore, RAF inhibitors allosterically perturb RAF autoinhibition, disrupting the N-terminal regulatory region (NTR) and C-terminal kinase domain (KD) interaction to facilitate the enhancement of RAS–RAF interaction [31]. LY3009120 has shown a higher anti-tumor effect on cells with *RAS* mutations as well as WT cells due to its higher binding potencies compared to monomer-selective inhibitors [24]. LY3009120 inhibits all RAF isoforms (A-Raf, B-Raf, and C-Raf) and occupies both RAF protomers with a similar affinity, resulting in various RAF homo- and heterodimers with a stabilized crystal conformation (αC-helix-in/DFG-out). Therefore, LY3009120 preforms the dimers with kinase activity and then blockades all RAF kinase catalytic activity.

Therefore, it is reported that pan-RAF binding potency is improved and that minimal paradoxical activation is induced in tumor cells harboring *RAF*^WT^ [20,23].

LY3009120 showed a similar significant inhibitory effect on the growth of three *RAS*-mutated equine and canine melanoma cell lines when compared to the effects observed in the reference cell line A375 (*BRAF* p.V600E). A possible LY3009120 mechanism acting here may be caused by the inhibiting of the downstream signaling of RAF dimers and MEK, thereby effectively inhibiting the transmission of the oncogenic signal from the *RAS*^mut^ melanoma cell lines (cRGO1, cRGO1.2, and MelDuWi).

Recently, the FDA approved two *KRAS*-G12C targeted inhibitors allowing direct RAS inhibition for specific isoforms. Further, currently over a dozen subsequent drugs are undergoing clinical evaluation targeting RAS directly or the closely related pathway proteins [32]. Accordingly, targeting the downstream molecules of RAS, such as the RAF kinases of the RAF-MEK-ERK pathway, can also effectively inhibit KRAS-mediated signaling pathways [33]. Pan-RAF inhibitors alleviate the suppression of negative feedback regulation on upstream RTK and RAS signals and facilitate other signaling pathways, such as the PI3K/AKT/mTOR pathway, which affects cell growth and proliferation [34]. The above-mentioned mechanisms may provide an explanation of why LY3009120 significantly affects cRGO1 (*NRAS*^mut^), cRGO1.2 (*NRAS*^mut^), and MelDuWi (*KRAS*^mut^). However, LY3009120 also showed significant effects on equine and canine wild-type MM cell lines, but these were less prominent. Interestingly, concentrations above 1 μM did not further decrease the metabolism of eRGO1 and cRGO4. In contrast, the higher LY3009120 concentrations induced an elevated metabolic activity, indicating a potential to trigger an alternative pathway activation similar to a feedback mechanism or paradoxical hyperactivation effect. Similarly, for the LY3009120-resistant equine melanoma cell line (eRGO6), the cause of resistance may be due to its accompanying molecular alterations, like the loss of tumor suppressor genes such as the *TP35*, *PTEN*, *CDKN2A*, *RB1*, *SPARC*, and PI3K/AKT pathway mutations, alterations in cell translational machinery (cytosolic ribosomal proteins (rProteins)) [35], and the dysregulation of the metabolism (HMOX1) [36]. Further, the compensatory mechanisms and adaptive reconnecting between the PI3K/AKT/mTOR and RAF/RAS/MEK/ERK pathways may also influence resistance mechanisms [37].

Another cause of resistance may be a low cellular dependence on the MAPK pathway. LY3009120 shows efficacy in cell lines displaying elevated baseline levels of pMEK and pERK [22,25]. Conversely, cell lines with lower levels of these phosphoproteins exhibit resistance to the blockade of the BRAF inhibitor [38].

Additionally, Cyclin D3 expression represents an MEK-independent pathway. Studies indicate that PRi + MEKi lacks activity in cell lines reliant on MAPK-independent survival [38,39]. Moreover, it is crucial to consider the *in vivo* tolerance of the effective dose, insufficient effect on target due to the short half-life or narrow therapeutic window of the drug, and the varied immune-related effects of the MEK block. These effects, unmeasurable *in vitro* or in patient-derived xenograft models, may significantly influence the treatment’s clinical activity.

LY3009120 appeared to have the highest anti-metabolic activity in MelDuWi and showed significant inhibition of the metabolism in cRGO1 and cRGO1.2. This indicates that LY3009120 may have a potential effect on *RAS* mutants of canine and equine melanoma cells with metabolic imbalances. The effects of *RAF/RAS* mutations on the melanoma metabolism include stimulating MYC and HIF1α signaling to promote glycolysis, as well as the inhibition of mitochondrial oxidative phosphorylation (OXPHOS) by repressing MITF and PGC1α, and promoting fatty acid synthesis. Therefore, glucose utilization and glycolytic flux are significantly reduced by inhibition of the RAS-RAF-MEK-ERK pathway [40]. *KRAS* mutations can lead to tumor-specific metabolic changes, thereby regulating oncogenic signaling networks and promoting tumor progression [41]. Mutant *KRAS* up-regulates the expression of the GLUT1 glucose transporter to promote glucose uptake. The glycolytic rate-limiting enzymes (hexokinase 1 and 2) also increase glycolytic activity [42]. Therefore, targeting metabolic enzymes may deserve further consideration for co-combination. However, in our study, the inhibitory effect on cell proliferation and biomass in MelDuWi was not more than that in eRGO1 (wild type). This may be because of the p.Q61H mutation with a high RAF kinase RBD (Ras-binding domain) affinity and the lowest intrinsic GTPase activity. Therefore, less upstream signaling will allow it to remain in the active GTP-bound state [43].

Furthermore, there has been a study using a pan-RAF inhibitor in combination with a MEK inhibitor (trametinib), evaluated in human melanoma cell lines, that achieved a significant inhibitory effect on cells growth [38]. In future perspectives, MEK inhibitors that also act on the MAPK/ERK pathway may have a similar potential. Currently, research continues to show actionable similarities and differences in the genetic mutational background and tumorigenic mechanisms in human, equine, and canine melanoma. This provides significant value for targeted therapeutic strategies in all three species. The possibility of applying targeted therapeutics developed in one species to another species is a basis for canine and equine models to become potential models of human melanoma disease. However, at the same time, the wild type also has an effect, suggesting that other explanations for cancer suppression pathways in different species await further investigation.

Our analyses have not yet involved the validation of pharmacodynamic mechanisms. However, we have conducted a series of *in vitro* experiments to prove the effectiveness of LY3009120. Following *in vivo* data and pharmacodynamic characterization remain for upcoming studies. In summary, pan-RAF and dimer inhibitor LY3009120, which was initially developed to target *BRAF*-resistant human melanoma cells, has demonstrated efficacy against *KRAS*/*NRAS*-driven and wild-type primary or metastatic equine and canine malignant melanoma cell lines, indicating that LY3009120 has promising potential for further *in vitro* or even *in vivo* application in horse and dog melanoma for both its phenotype and mechanism of efficacy as well as drug resistance.

## Figures and Tables

**Table 1:** Clinical information on the equine and canine melanoma cell lines: variety, age, gender, location, and diagnosis.

Patient	Cell Line	Breed	Age (y)	Sex	IHC	Diagnosis	Location	Tumor Type
Canine								
1	Mel1268	Mix	10	f	_	OMM	Oral cavity	Primary MM
2	Mel0910	Yorkshire Terrier	15	f	_	Cutaneous MM	Ear	Primary MM
3	cRGO1	Mix	11	f	Melan-A +, PNL2 +, CD146	Malignant predominantly amelanotic melanoma	Back-end hard palate	Primary OMM
4	cRGO1.2 *				Melan-A +, S100 -, Vimentin ++, MMP2 +++, MMP9 +		Left mandibular LN	LN metastasis
5	cRGO4	Golden Retriever	9	m	Melan-A +, PNL2 +, CD146	OMM	Right mandible	Primary MM
6	cRGO6	Weimaraner	10	f	HMB45 -, PNL2 ++	Amelanotic OMM (III (T2N1M0))	Multiple skin lesions	Cutaneous metastasis
Equine								
7	MelDuWi	Andalusian Grey horse	15	m	_	Cutaneous EMM	Skin, lip, eye, penis, and anus	Primary EMM
8	eRGO1	Icelandic horse	16	m	_	Multiple cutaneous EMM	Under the tail	Primary EMM
9	eRGO6	Grey horse	12	f	_	Metastasizing melanoma	Liver	Liver metastasis

LN: lymph nodes; OMM: oral malignant melanoma; EMM: equine malignant melanoma; MM: malignant melanoma; m: male; f: female; +: weakly positive; ++: positive; +++: strong positive; -: negative; _: not tested; and *: resistant to irradiation (0–6 Gy) [26,28]. Origin of the data from the cell culture stock of the Clinic for Horses and Small Animal Clinic, University of Veterinary Medicine Hannover, Foundation, Germany, and the University of Veterinary Medicine, Vienna, Austria [26].

**Table 2:** Primers of analysis *BRAF*, *NRAS*, and *KRAS* exon mutations.

Species	Gene	Primer Sequences (5′-3′)		Template	Fragment	Tm [°C]	Ta [°C]	Transcript
Canine ^a^	*BRAF* exon 15	F	cacgccaagtcaatcatccacaga	cDNA	228 bp	62.7	55.3	*BRAF*-202 ENSCAFT00000006306.4
		R	cccaaatgcgtatacatctgactgg			63.0		
	*NRAS* exon 2	F	agcttgaggttcttgctggtgtga	cDNA	474 bp	62.7	59.3	*NRAS*-202
								ENSCAFT00845046556.1
		R	tgtctggtcttggctgaggtttca			62.7		
Equine ^b^	*BRAF* exon 11	F	tccctttcaggcatagggta	gDNA	247 bp	57.3	53.9	*BRAF*-206 ENSECAT00000005257.4
		R	tgacatgtgacaaggtcattgtat			57.6		
	*BRAF* exon 15	F	tcataatgcttgctctgataagaaa	gDNA	236 bp	56.4	59.3	*BRAF*-206 ENSECAT00000005257.4
		R	cagcatctcagggtccaaa			56.7		
	*NRAS* exon 2	F	gtactgtagatgtggctcgc	gDNA	224 bp	59.4	62.3	*NRAS*-201 ENSECAT00000014647.2
		R	acggaagaaagagaggtgga			57.3		
	*KRAS* exon 2	F	ccagactgtgtttctcccttc	gDNA	249 bp	59.8	61.1	*KRAS*-203 ENSECAT00000020767.2
		R	caattactcctccatgtcaattt			55.3		

F: forward primer; R: reverse primer; Tm: melting temperature; and Ta: annealing temperature. ^a^: primers are intron spanning. Primers design according to Fowles JS et.al [4]. ^b^: Primer of equine *NRAS* exon 2 design from Primer3, the rest of primers design according to Jiang Lin et.al [9].

**Table 3:** The genotypes of the human, canine, and equine melanoma cell lines by DNA sequencing.

	Mutation Genotypes									
	Human	Equine			Canine					
Gene	A375	MelDuWi	eRGO1	eRGO6	Mel1268	Mel0910	cRGO1	cRGO1.2	cRGO4	cRGO6
*BRAF* exon 15	p.V600E	-	-	-	-	-	-	-	-	-
*BRAF* exon 11	-	-	-	-	-	-	-	-	-	-
*NRAS* exon 2	-	-	-	-	-	-	p.G13R	p.G13R	-	-
*NRAS* exon 3	-	-	-	-	-	-	-	-	-	-
*KRAS* exon 2	-	p.Q61H	-	-	-	-	-	-	-	-
*KIT* exon 11	-	-	-	-	-	-	-	-	-	-

Human data were tested by a targeted sequencing panel covering 50 cancer-relevant genes (Cancer Hotspot Panel V2). -: wild Type. MelDuWi (CAA>CAT, p.Gln61His) is homozygous SNV. VAF: allele frequency. cRGO1 and cRGO1.2 (NM_001287065.1:g.52418151 G>C) are heterozygous SNV, cRGO1 (VAF: 28) and cRGO1.2 (VAF: 29).

## Data Availability

Data are available upon direct request.

## Supplementary Materials

The following supporting information can be downloaded at https://www.mdpi.com/article/10.3390/genes15020202/s1: Table S1: Summary of molecular/genetic evaluation to date in canine and equine melanomas [4,5,6,7,44,45,46,47,48,49,50]; Table S2: Clinical characteristics, pathological differentiation, and mutation genes for human, equine, and canine melanoma [3,10,49,50,51]; Figure S1: Morphology of all human, canine, and equine melanoma cell lines by microscopy after 72 h seeding. (400× magnification); Figure S2: Proliferation, biomass, and metabolic activity in ten melanoma cell lines after 48 h and 72 h exposure to LY3009120.
