# Supplementary material for: Cross-Species Comparison of the Pan-RAF Inhibitor LY3009120’s Anti-Tumor Effects in Equine, Canine, and Human Malignant Melanoma Cell Lines
Source: Genes (Basel). 2024 Feb 3;15(2):202. doi: 10.3390/genes15020202 (PMC10887541; doi:10.3390/genes15020202)
Supplement: Supplementary file 1 [file genes-15-00202-s001.zip › Supplement 1 Table S1 ,S2 , Figure S1 and Figure S2.pdf]

Supplement Table S1. Summary of molecular/genetic evaluation to date in canine and equine melanomas.

Gene（Canine）/Total%	Site/Total %	Investigative approach	Specimen	Affected/ Examined	Mutation	Reference
NRAS 25/311 (8.0%)	Mucosal 11/115 (9.6%)	*NRAS* Exon 2 sequencing	Tumors and cell lines (Jones)	1/16 (6.25%)	Q61R	Fowles JS, 2015 [4]
		Sanger sequencing	Tumors and cell lines (UCDK9M5 and Jones)	4/35 (11.4%)	Q61	Wei BR, 2016 [44]
		WES	Tumors	7/65 (11.0%)	4 cases with Q61H/R/K<br>3 cases with G12A/D	Wong K, 2019 [7]
	Digit 11/169 (6.5%)	Sanger sequencing	Tumors	2/83 (2.4%)	Exon 2 Codon 12	Conrad D, 2022
		Sanger sequencing	Tumors	9/86 (10.5%)	Exon 3 Codon 61	
	Skin	Codon 61 sequence	Tumors	2/16 (12.5%)	Exon 2 Codon 61	Mayr B, 2003 [45]
	Unknown	PCR of multiple codons	Tumors	1/11 (9.1%)	Exon 1 Codon 22	Murua Escobar H, 2004 [46]
BRAF 5/272(1.8%)	Mucosal 4/180 (2.2%)	Orthologous codon 599, exon 15, clone and sequence	Tumor and Cell lines	0/68 (0%)	All absent	Fowles JS, 2015<br>Wei BR, 2016<br>Shelly S, 2005 [47]
		BRAF exon 15 sequencing	Tumors	2/47 (4.3%)	V450E	Mochizuki H, 2015 [48]
		WES	Tumors	2/65 (3.1%)	G457A, D582G	Wong K, 2019
	Skin	*BRAF* exon 15 sequencing	Tumors	1/6 (17%)	V450E	Mochizuki H, 2015

	Digit	Digital droplet PCR (ddPCR)	Tumors	0/86 (0%)	BRAF V595E absent	Conrad D, 2022
KRAS 27/206 (13.1%)	Digit	Sanger sequencing	Tumors	22/86 (25.6%)	Exon 2 codons 12&13	Conrad D, 2022
		Sanger sequencing	Tumors	2/55 (3.6%)	Exon 3 codon 61	
	Mucosal	WES	Tumors	3/65 (4.6%)	G12D/V, G13D	Wong K, 2019
c-kit 16/70 (22.9%)	Digit	Sanger sequencing	Tumors	16/70 (23%)	Exon 11 mutations Single nucleotide polymorphism	Conrad D, 2022

**Gene（Equine）/Total%**	**Site**	**Investigative approach**	**Specimen**	**Affected/ Examined**	**Mutation**	**Reference**
NRAS 5/60 (8.3%)	Cutaneous	WES	Tumors	1/32 (3.1%)	Q61R	Wong K, 2019
	Mucosal-like/ Mucocutaneous			4/28 (14%)	3 cases with Q61R 1 case with G12A	
BRAF 2/28 (7.1%)	Mucosal-like/ Mucocutaneous			2/28 (7.1%)	V594E, P321L and S56L in vulvar tumor HD0021a	
KIT 1/28 (3.6%)				1/28 (3.6%)	S339F	
KRAS 0/62 (0%)	Cutaneous /Mucosal-like/ Mucocutaneous			0/62 (0%)	Wild type	

Table update based on David Conrad et al [5], R Mark Simpson et al [6], and Kim Wong et al [7].
WES: Whole exome sequencing.

Supplement Table S2. Clinical characteristics, pathological differentiation, and mutation genes for human, equine, and canine melanoma.

	**Clinical characteristics**		**Pathological differentiation**		**Common mutation genes**	**Median survival time of MM (month)**
	**Main classification**	**Main Location sites**	**HE**	**IHC**		
Human*	Superficial spreading/Nodular/Lentigo/ Acral lentiginous melanoma	Back/ legs	Animal type/ Simulating naevi type/ Epithelioid type	MITF/S100/Melan A/HMB 45/SOX10/PRAME	*BRAF*/*RAS*/*NF1*/*c-kit*/*none* [49]	60 months [50]**
Equine	Melanocytic naevus/Discrete derma/ dermal melanomatosis/Anaplastic MM	Tail/perianal/lips/eyelids		S100/NSE/PNL2/HMB 45/PCNA/Ki-67/T-311/RACK1/CD44	*STX17*/*NR4A3*/*NRAS*/*DPF3* [3, 51]	-
Canine	Oral/Cutaneous /Ocular /Subungual	Oral		HMB 45/Melan A/S100 /MITF/TRP2/PNL2	*NRAS*/*KRAS*/*c-Kit*/*PTPRJ*	6 months (Oral [10])

*** Unusual Moles that Indicate Human Melanoma: simply distinguish from Asymmetry, Border, Color, Diameter, and Evolving**
**** Combination immunotherapy (ipilimumab/nivolumab)**
-: not found; HE:Hematoxylin and Eosin stain; IHC: Immunohistochemistry; MM: Malignant melanoma; PRAME: Preferentially expressed antigen in melanoma.

Supplement Figure S1. Morphology of all human, canine, and equine melanoma cell lines by microscopy (400x magnification) after 72 hours seeding.

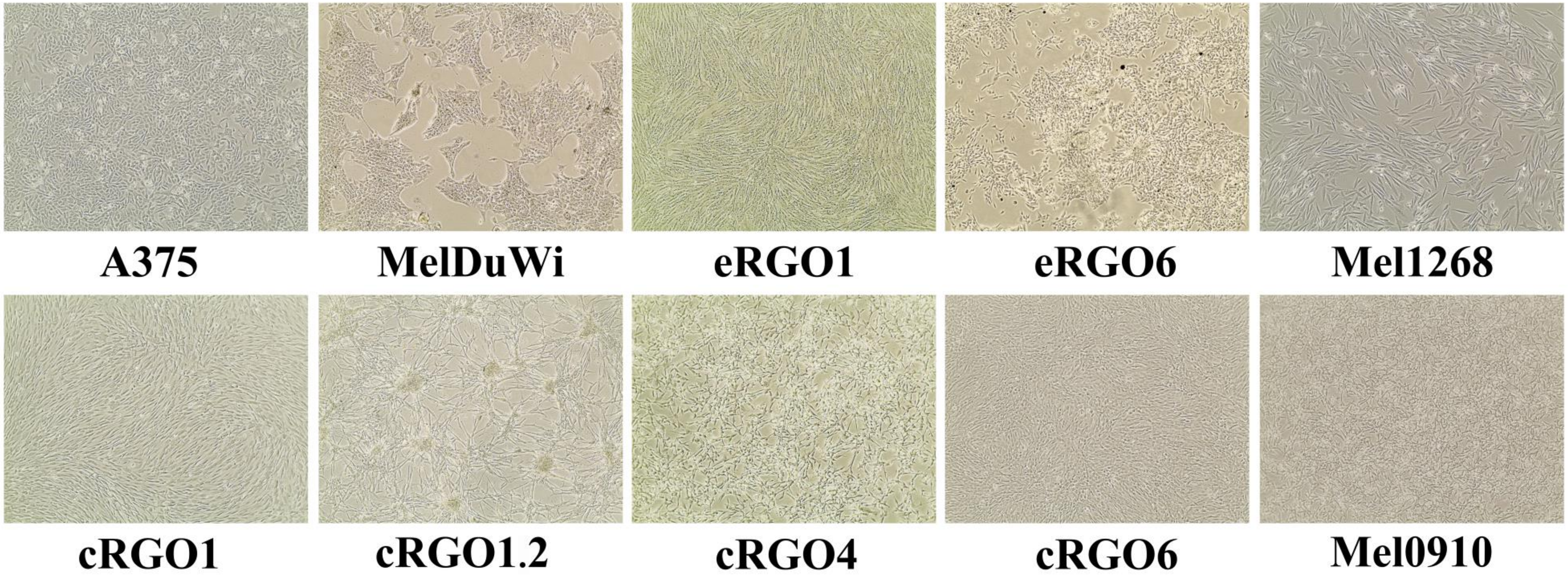

A375 (derived from a skin primary human malignant melanoma, carrying the *BRAF* V600E mutation); MelDuWi (equine cutaneous malignant melanoma, harbor the *KRAS* p.Q61H mutation); eRGO1(equine cutaneous malignant melanoma); eRGO6 (equine liver metastasis melanoma); Mel1268 (canine primary oral malignant melanoma); Mel0910 (canine primary cutaneous malignant melanoma); cRGO1 (canine primary predominately amelanotic malignant melanoma, carry *NRAS* p.G13R); cRGO1.2 (canine left mandibular lymph node metastasis, carry *NRAS* p.G13R); cRGO4 (canine primary oral malignant melanoma); cRGO6 (canine amelanotic oral malignant melanoma); Compared to other melanoma cell lines, metastases-derived cell line cRGO1.2 displayed a more fibroblastic, bipolar and elongated morphology.

Supplement Figure S2. Proliferation, biomass, and metabolic activity in ten melanoma cell lines after 48h and 72h exposure to LY3009120.

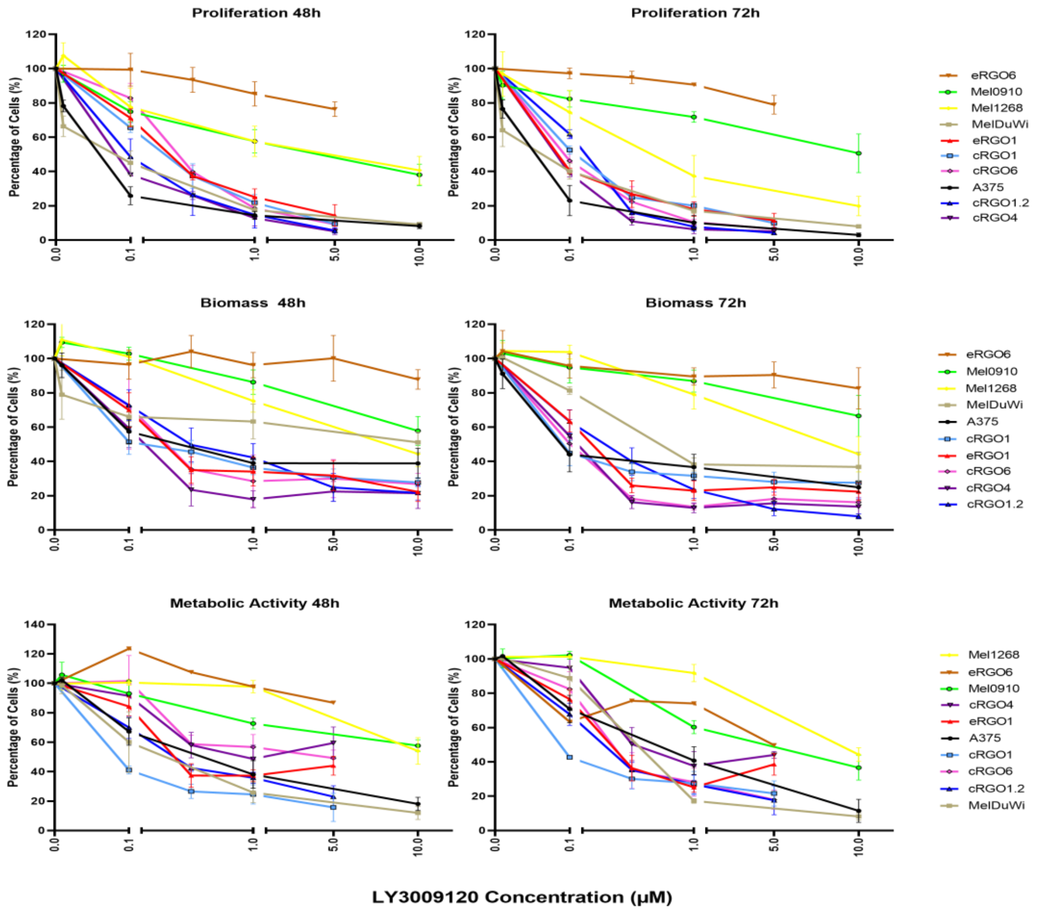

Except for liver metastasis equine melanoma cells eRGO6, LY3009120 acted dose-dependent and time-dependent in all melanoma cell lines. cRGO1.2, cRGO4, cRGO6, and eRGO1 showed a higher inhibition as the positive control A375 in terms of the reduced biomass and metabolic activity effect at 1 µM. cRGO1.2 and cRGO4 showed a higher inhibition as the positive control A375 in terms of the antiproliferation effect at 1 µM. In metabolic activity, eRGO1 and cRGO4 showed a rebound after the high concentration of 1 µM.
